# Supplementary material for: TMT-Based Quantitative Proteomics Analysis Reveals Differentially Expressed Proteins between Different Sources of hMSCs
Source: Int J Mol Sci. 2023 Aug 31;24(17):13544. doi: 10.3390/ijms241713544 (PMC10488246; doi:10.3390/ijms241713544)
Supplement: Supplementary file 1 [file ijms-24-13544-s001.zip › ijms-2548015-supplementary.pdf]

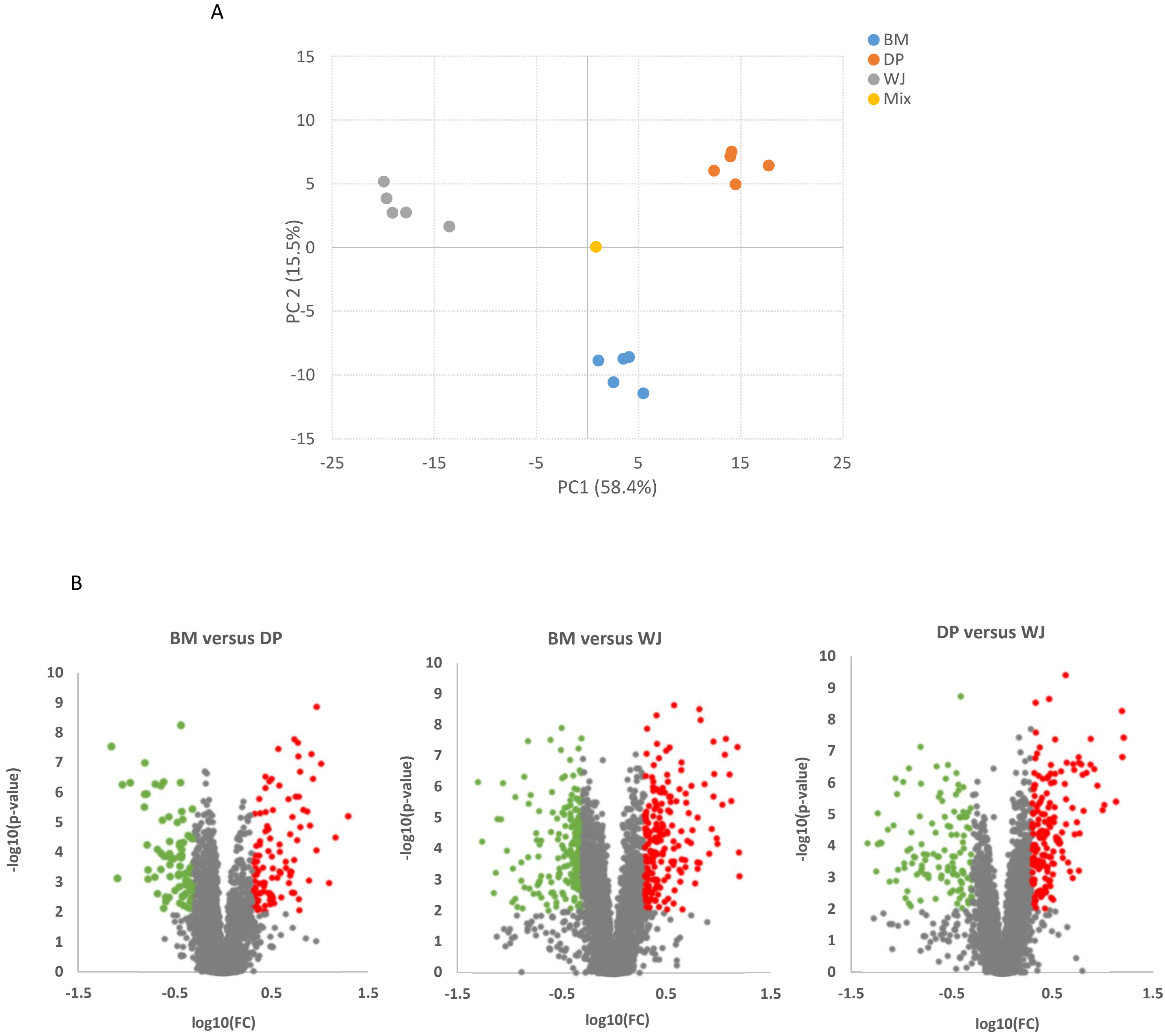

Supplementary Figure S1. (**A**) Principal component analysis (PCA) of protein abundances for all biological replicates of different sources (WJ, BM and DP) and Mix for the control. (**B**) Volcano plots of differently proteins showing the changes in proteins for BM, WJ and DP hMSCs. The DAPs were identified based on t test with p-value less than 0.05 and with a fold change >2 (red) or <0.5 (green).

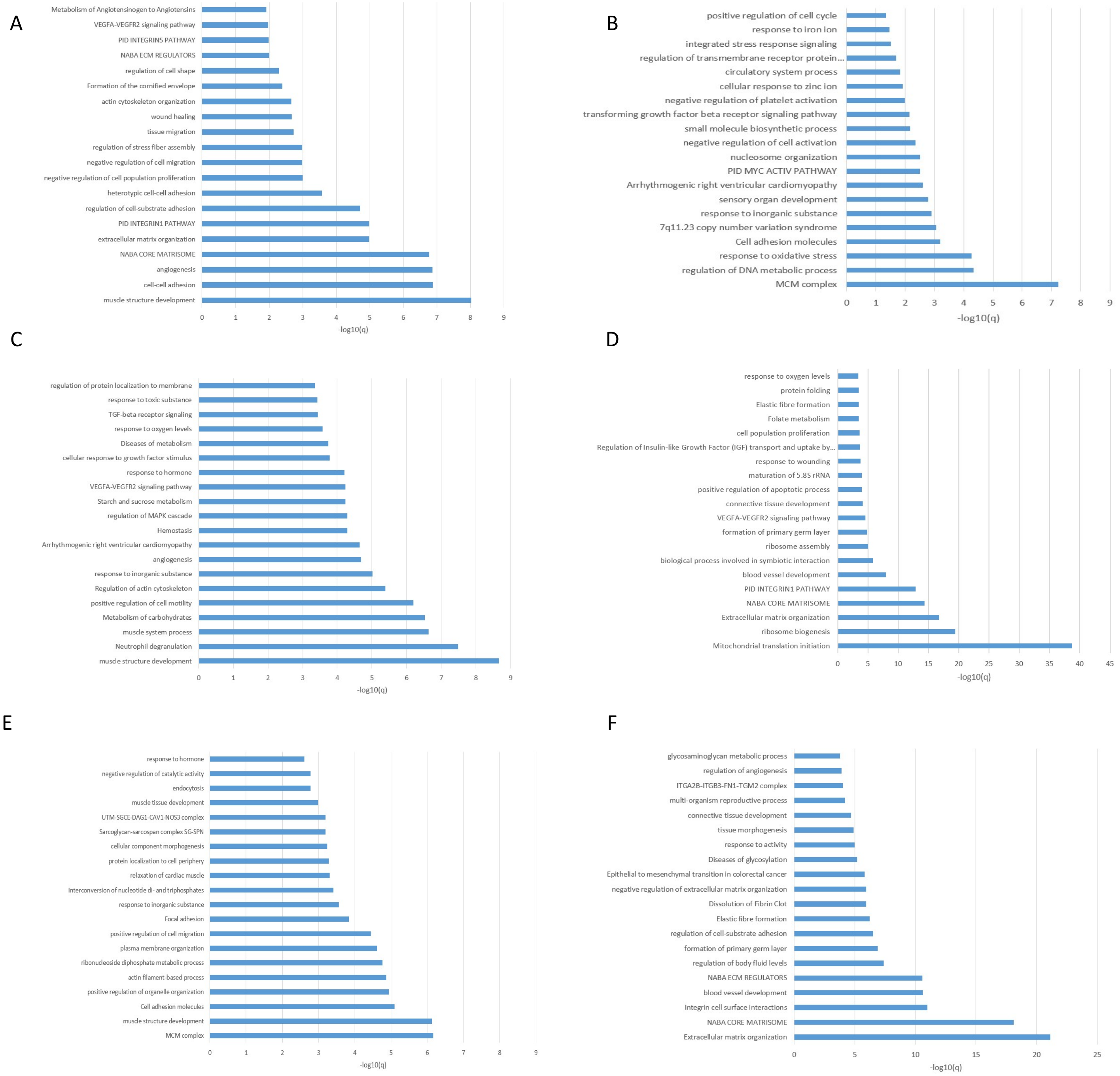

Supplementary Figure S2. GO (biological process) analysis using pairwise comparison of the proteins significantly up-regulated in BM *vs* DP (**A**), BM *vs* WJ (**C**), DP *vs WJ* (**E**) and down-regulated in in BM *vs* DP (**B**), BM *vs* WJ (**D**), DP *vs WJ* (**F**). q-values are calculated using the Benjamini-Hochberg procedure to account for multiple testing. Kappa scores are used as the similarity metric when performing hierarchical clustering on the enriched terms, and sub-trees with a similarity of > 0.3 are considered a cluster.

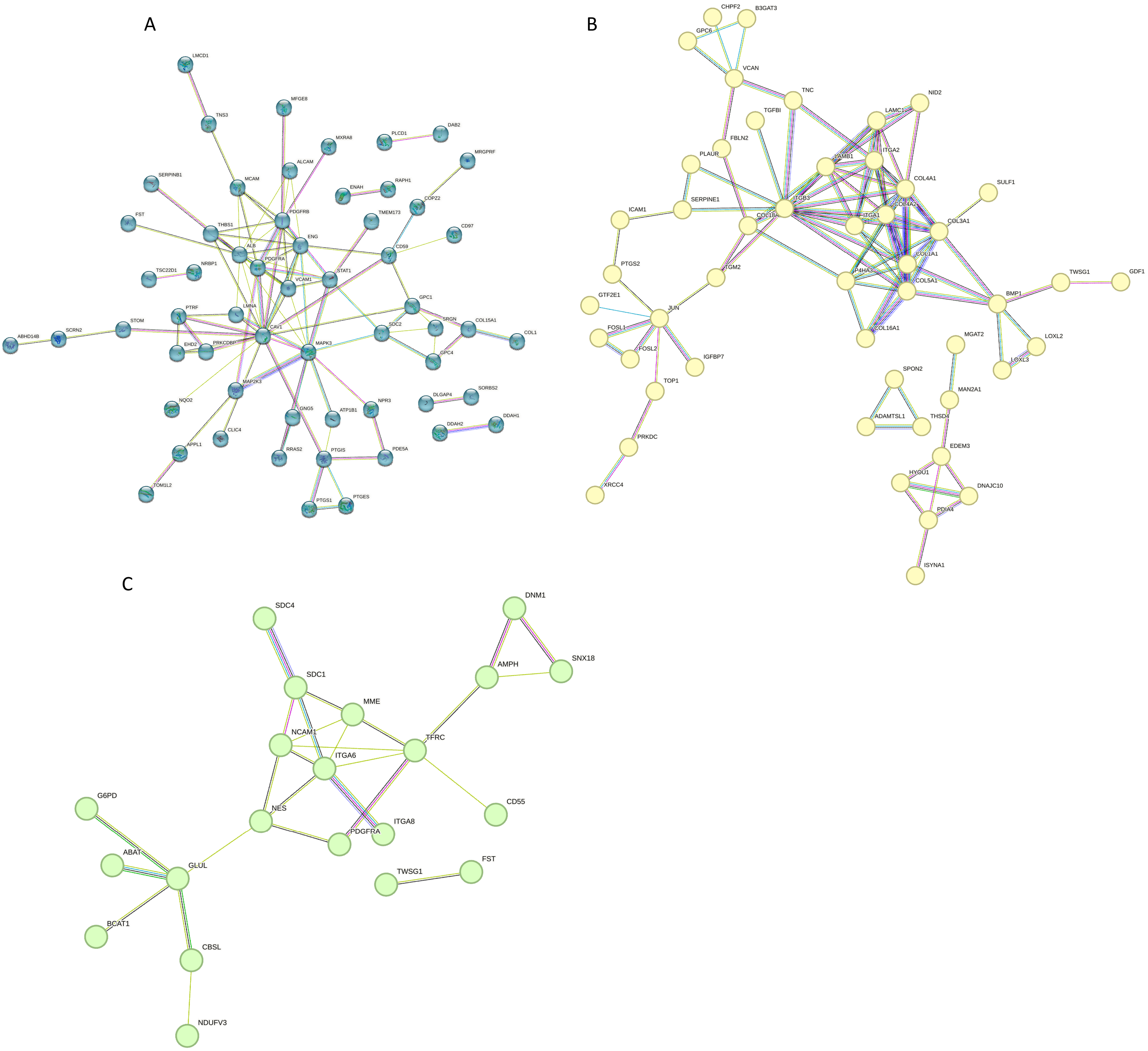

Supplementary Figure S3. Protein-protein interaction analysis of up-regulated proteins in BM (**A**), WJ (**B**), DP (**C**) hMSCs samples using STRING database (https://string-db.org) by setting up the parameters as *Homo sapiens* and combined confidence score greater than 0.4. WJ produced more ECM proteins and ECM-affiliated proteins. BM-MSCs display enhanced differentiation and paracrine communication capabilities. DP-MSC appeared to promote exosome production.

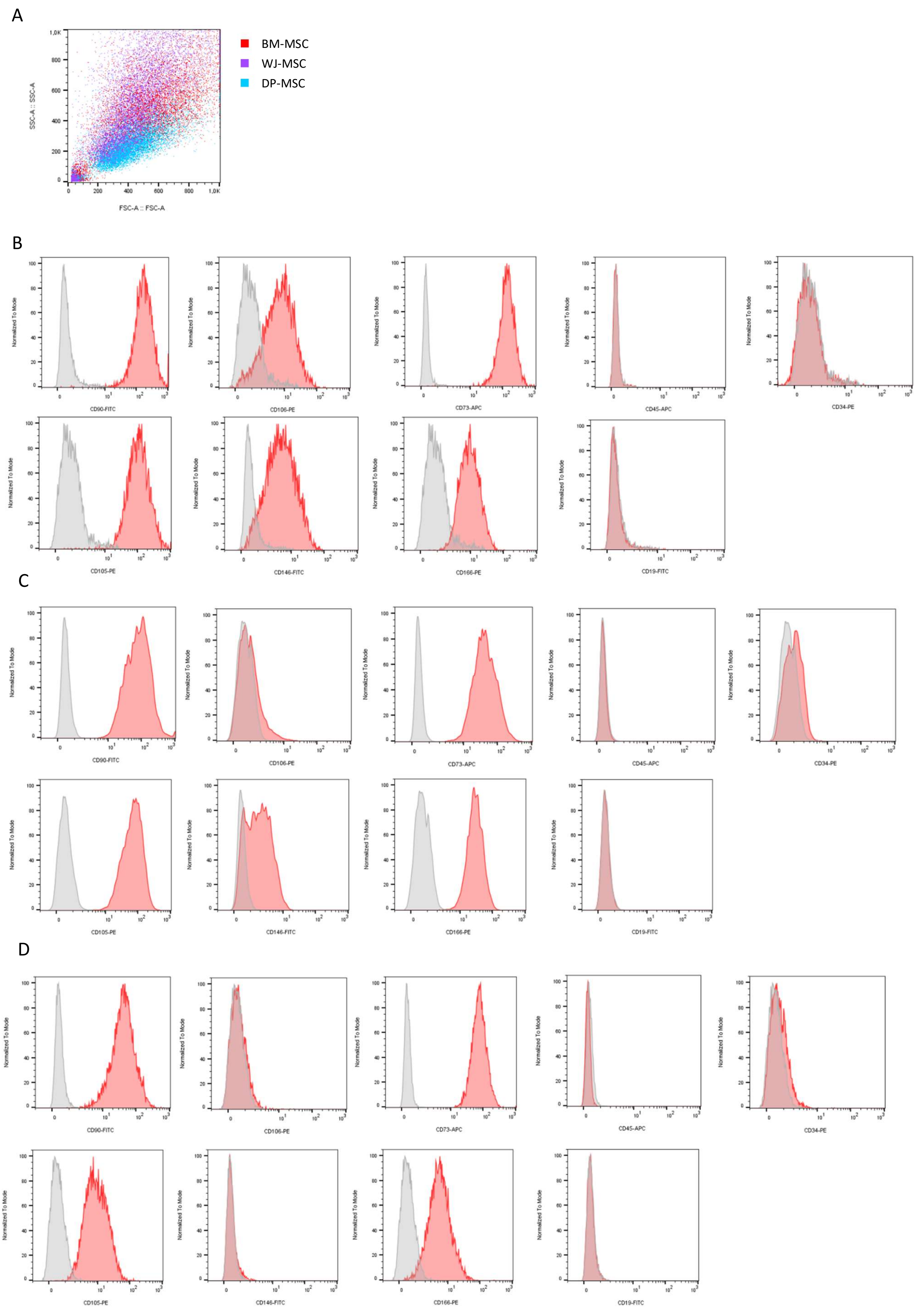

Supplementary Figure S4. Flow cytometric analysis of MSCs surface markers. (**A**) Gating strategy used for the identification of BM-MSC (in red), WJ-MSC (in purple) and DP-MSC (in blue). Same number of events was counted for different sources : BM-MSC (**B**), WJ-MSC (**C**) and DP-MSC (**D**). Positive markers (CD90, CD105 and CD73), negative markers (CD45, CD34 and CD19) and variants markers (CD106, CD146 and CD166) are shown in red, isotypes are shown in grey.
